# Connecting complex networks to nonadditive entropies

**DOI:** 10.1038/s41598-020-80939-1

**Published:** 2021-01-13

**Authors:** R. M. de Oliveira, Samuraí Brito, L. R. da Silva, Constantino Tsallis

**Affiliations:** 1 Departamento de Física Teórica e Experimental, Federal University of Rio Grande do Norte, Natal, RN 59078-900 Brazil; 2 International Institute of Physics, Federal University of Rio Grande do Norte, Natal, RN 59070-405 Brazil; 3 National Institute of Science and Technology of Complex Systems, Rio de Janeiro, Brazil; 4 Centro Brasileiro de Pesquisas Físicas, Rua Xavier Sigaud 150, Rio de Janeiro, RJ 22290-180 Brazil; 5 Santa Fe Institute, 1399 Hyde Park Road, New Mexico, 87501 USA; 6 Complexity Science Hub Vienna, Josefstaedter Strasse 39, Vienna, 1080 Austria

**Keywords:** Physics, Statistical physics, thermodynamics and nonlinear dynamics, Complex networks, Statistical physics

## Abstract

Boltzmann–Gibbs statistical mechanics applies satisfactorily to a plethora of systems. It fails however for complex systems generically involving nonlocal space–time entanglement. Its generalization based on nonadditive *q*-entropies adequately handles a wide class of such systems. We show here that scale-invariant networks belong to this class. We numerically study a *d*-dimensional geographically located network with weighted links and exhibit its ‘energy’ distribution per site at its quasi-stationary state. Our results strongly suggest a correspondence between the random geometric problem and a class of thermal problems within the generalised thermostatistics. The Boltzmann–Gibbs exponential factor is generically substituted by its *q*-generalisation, and is recovered in the $q=1$ limit when the nonlocal effects fade away. The present connection should cross-fertilise experiments in both research areas.

## Introduction

Boltzmann–Gibbs (BG) statistical mechanics constitutes one of the pillars of contemporary theoretical physics. As such is has uncountable successes for a great variety of physical systems. However, when the system constituents have a generically nonlocal space–time entanglement, this theory does not apply. Such is the case already pointed in 1902 by Gibbs himself, namely when the standard partition function diverges, e.g., gravitation. It is in this context that it was proposed in 1988^1^ the generalisation—hereafter referred to as *nonextensive statistical mechanics*—of the BG theory based on nonadditive entropies, namely $S_q = k \frac{1-\sum _i p_i^q}{q-1}$, which recovers $S_{BG}=-k \sum _{i} p_i \ln p_i$ in the $q\rightarrow 1$ limit. The composition of two probabilistically independent systems *A* and *B* yields straightforwardly $S_q(A+B)/k= [S_q(A)/k] + [S_q(B)/k] +(1-q) [S_q(A)/k][ S_q(B)/k]$. As we see, the BG entropic additivity is recovered when $q=1$. The fundamental advantage associated with $q \ne 1$ is that, for strongly correlated systems, it enables, as illustrated in^2^, the preservation of the *extensivity* of the thermodynamic entropy, mandated in all circumstances by the Legendre structure of classical thermodynamics.

In parallel with the above, the study of complex networks has been intensified around the world^3–7^. Networks can be found everywhere. Society is formed by humans linked through relationships. The Internet is a set of devices communicating with each other. The brain is formed by neurones communicating through synapses. All these completely different systems can be translated onto a simple set of *nodes* (or *sites*) and *edges* (or *links*) obeying some connection rule, and the tools of network science can be successfully used to study them. Typical applications of this area can be found in classical and quantum internet^8,9^, medicine^10,11^, neuroscience^12^, and sociology^13,14^. It was thought, during more than a decade, that most of real networks were purely scale-free meaning that the distribution of the number of links in the network follows a power-law distribution. It was recently argued that most real networks are not pure scale-free^15^, paving the way for new possibilities to describe them.

During the initial years, network science and nonextensive statistical mechanics were seen as completely different areas. But meaningful connections started in 2005^16–22^. It is nowadays known that the degree distribution of asymptotically scale-free networks at the thermodynamic limit is of the form $P(k) \propto e_q^{-k/\kappa }$, where the *q*-exponential function is defined as $e^{z}_q\equiv [1 + (1-q)z]^{\frac{1}{1-q}}$ ($e_1^z=e^z$). This form, more precisely $p_q(\varepsilon _i) = e_q^{-\beta _q\,\varepsilon _i}/Z_q$, optimizes the entropy $S_q$ under appropriate canonical constraints, $\varepsilon _i$ being the site energy and $\beta _q$ the inverse temperature; the BG weight is recovered at the $q\rightarrow 1$ limit. This thermostatistical approach has been successfully applied in a wide diversity of areas, such as long-range-interacting Hamiltonian systems^23^, vortices in type II supercondutors^24^, cold atoms^25^, granular matter^26^, high-energy physics experiments on Earth^27^ and observations in the outer space^28,29^, civil engineering^30^, and for predicting COVID-19 peaks around the world^31,32^.

**Figure 1:**
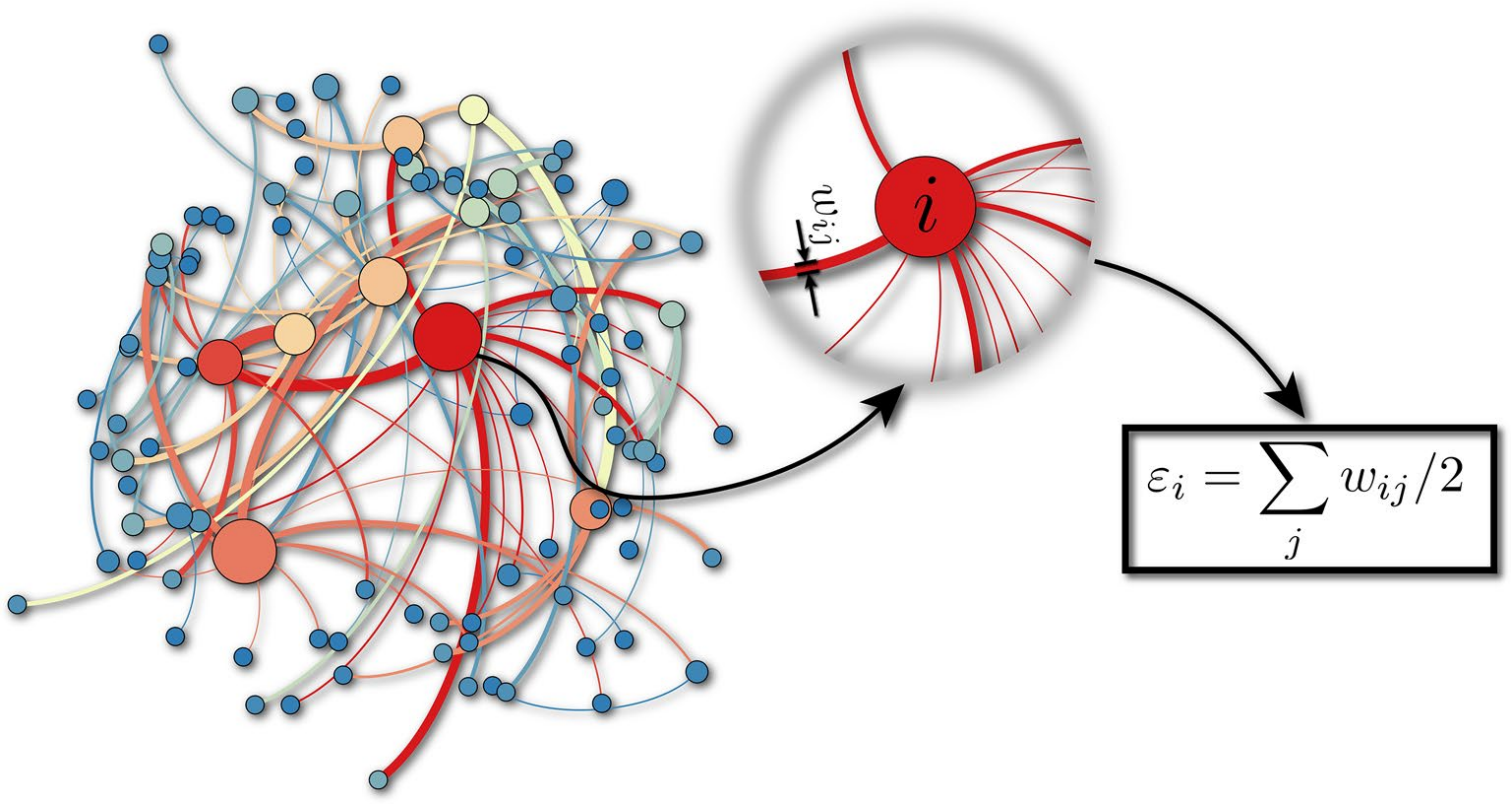
Sample of a $N=100$ network for $(d,\alpha _A,\alpha _G,\eta ,w_0) = (2,1,5,1,1)$. As can be seen, for this choice of parameters, hubs (highly connected nodes) naturally emerge in the network. Each link has a specific width $w_{ij}$ and the total energy $\varepsilon _i$ associated to the site *i* will be given by half of the sum over all link widths connected to the site *i* (see zoom of site *i*).

In this work, we introduce and study a geographically located *d*-dimensional network model. One of the main characteristics of this model is the possibility to control the long/short range nature of the interactions between the sites. BG statistics completely fails to describe systems that interact at long-range, and many theories have been proposed to approach this regime. The present model introduces a new property for this class of systems. In addition to the fact that Euclidean distances ($d_{ij}$) between the sites are relevant, it also takes into account the weights ($w_{ij}$) of the links (see Fig. 1) and associates them to the ‘energy’ ($\varepsilon _i$) of each site. Due to that new ingredient, we could compute the energy distribution of the ever growing network. This distribution turns out to have the functional form of the *q*-generalised BG distribution for nonextensive systems, based on nonadditive entropy. These numerical results strongly suggest a neat correspondence between the random network geometrical problem and a particular thermostatistical problem within the generalised theory.

## The model

Our growing *d*-dimensional network starts with one site at the origin. We then stochastically locate a second site (and then a third, a fourth, and so on up to N) through a probability $p(r) \propto 1/r^{d+ \alpha _G} \;\;\; (\alpha _G > 0)$, where $r\ge 1$ is the Euclidean distance from the newly arrived site to the center of mass of the pre-existing cluster; $\alpha _G$ is the *growth* parameter and $d=1,2,3$ is the dimensionality of the system (large $\alpha _G$ yields geographically concentrated networks).

The site $i=1$ is then linked to the site $j=2$. We sample a random number $w_{ij}$ from a distribution *P*(*w*) that will give us the corresponding link weight. Each site will have a *total energy* $\varepsilon _i$ that will depend on how many links it has, noted $k_i$, and the widths $\{ w_{ij}\}$ of those links. At each time step, the site *i* only has access to its *local energy* $\varepsilon _i$ defined as:

1 $$\begin{aligned} \varepsilon _i \equiv \sum _{j=1}^{k_i} \frac{w_{ij}}{2} \;\;\;(w_{ij} \ge 0) \end{aligned}$$

The value of $\varepsilon _i$ will directly affect the probability of the site *i* to acquire new links. Indeed, from this step on, the sites $i =3,4, \ldots$ will be linked to the previous ones with probability

2 $$\begin{aligned} \Pi _{ij}\propto \frac{\varepsilon _{i}}{d^{\,\alpha _A}_{ij}} \;\;(\alpha _A \ge 0)\,, \end{aligned}$$

where $d_{ij}$ is the Euclidean distance between *i* and *j*, where *j* runs over all sites linked to the site *i*. The *attachment* parameter $\alpha _A$ controls the importance of the distance in the preferential attachment rule (2). When $\alpha _A \gg 1$ the sites tends to connect to close neighbours, whereas $\alpha _A \simeq 0$ tends to generate distant connections all over the network. Notice that, while the network size increases up to *N* nodes, the variables $k_i$ and $\varepsilon _i$ (number of links and *total energy* of the *i*-th node; $i=1,2,3 \dots ,N$) also increase in time (see Fig. 1 for a sample of the ever growing network).

If we consider the particular case $P(w)=\delta (w-1)$, where $\delta (z)$ denotes the Dirac delta distribution, Eq. (2) becomes $\Pi _{ij}\propto k_{i}/d^{\,\alpha _A}_{ij} \;\;(\alpha _A \ge 0)\,$, thus recovering the usual preferential attachment rule. Consequently, the present model recovers the one in^19–21^ as a particular instance. Note that, if we additionally consider the particular case $\alpha _A=0$, we recover the standard Barabási–Albert model with $\Pi _i \propto k_i$^5,6^.

We are considering here the case where *w* is given by the following stretched-exponential distribution:

3 $$\begin{aligned} P(w) = \frac{\eta }{w_0\,\Gamma \left( \frac{1}{\eta }\right) } e^{-(w/w_0)^{\eta }} \;\;(w_0>0;\, \eta > 0)\,, \end{aligned}$$

which satisfies $\int _0^\infty dw\,P(w)=1$. As particular cases of Eq. (3) we have: $\eta = 1$, which corresponds to an exponential distribution, $\eta = 2$, which corresponds to a half-Gaussian distribution, and $\eta \rightarrow \infty$, which corresponds to an uniform distribution within $w \in [0,w_0]$.

**Figure 2:**
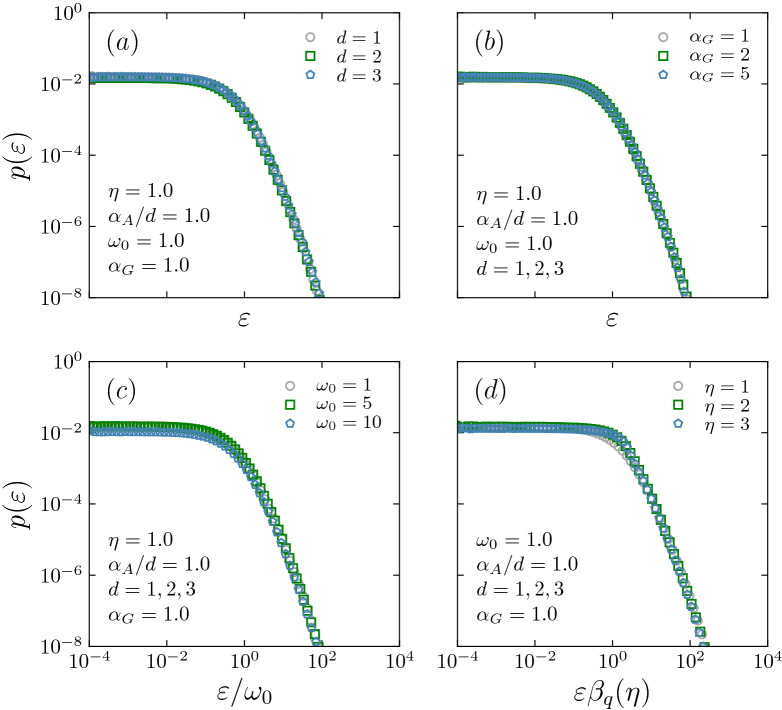
In these plots we show $p(\varepsilon )$ for typical values of *d* (**a**), $\alpha _G$ (**b**), $w_0$ (**c**) and $\eta$ (**d**). (**a**) By fixing $(\alpha _G,\eta ,w_0,\alpha _A/d) = (1,1,1,1)$ the dimensionality *d* does not modify $p(\varepsilon )$. (**b**) By fixing $(\eta ,w_0,\alpha _A/d) = (1,1,1)$, $\alpha _G$ has no influence on $p(\varepsilon )$. (**c**) We show that variations of $w_0$ yield a $p(\varepsilon )$ which remains invariant when expressed in terms of $\varepsilon /w_0$. (**d**) We show that for variations of $\eta$ the curves of $p(\varepsilon )$ versus $\varepsilon \beta _q(\eta )$ collapse once again. For simplicity, the values of the fixed variables were set equal to unity, but the results remain independent from this choice. The numerical precision of all the collapses is verified to be quite impressive. Very tiny discrepancies might be due to the fact that both *N* and the number of realisations are finite, and/or to high-order metric-topological terms. The simulations were averaged over $10^3$ realisations for $N=10^{5}$.

## Results

Our focus here is to analyse the energy distribution $p(\varepsilon )$ of the $N \gg 1$ network. We have in fact analyzed a large amount of typical cases in the space $(d, \alpha _A, \alpha _G, w_0,\eta )$, and have systematically found the same results for $d=1,2,3$ within the intervals $(\alpha _A/d \in [0,10]; \alpha _G \in [1,10]; w_0 \in [0.5, 10]; \eta \in [0.5,3])$. Similarly to previous works^16,19–21^, $p(\varepsilon )$ does not depend on $\alpha _G$; also, it does not depend independently on *d* and $\alpha _A$, but only, remarkably, on the ratio $\alpha _A/d$; $p(\varepsilon )$ also depends on $w_0$ and $\eta$ (see Fig. 2a–d). Because of these features, and without loss of generality, we have once for ever fixed $\alpha _G = 1$, and $d=2$. The simulations were done for $10^3$ realisations of size $N=10^{5}$, which was verified to be enough for observing the asymptotic distribution $p(\varepsilon )$ with high precision.

**Figure 3:**
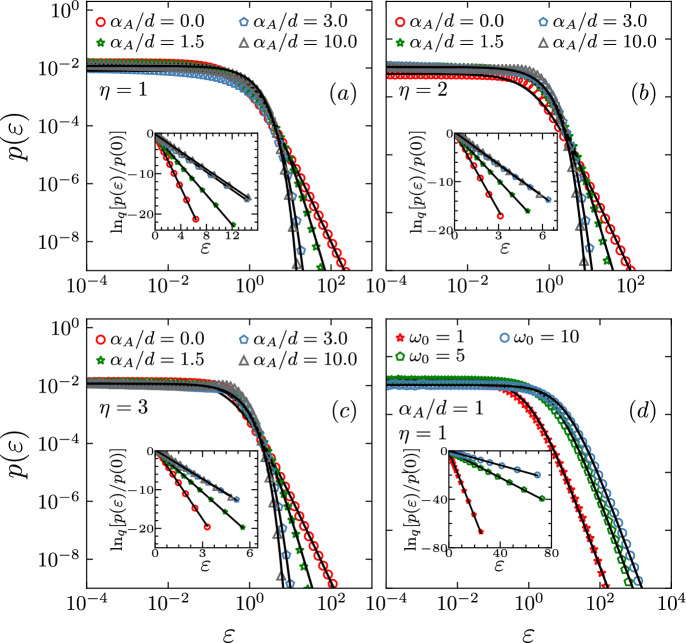
In these plots we show the variations of $p(\varepsilon )$ for fixed values $\eta = 1$ (**a**) , $\eta = 2$ (**b**) and $\eta = 3$ (**c**), for $\alpha _A/d = 0,1.5, 3,10$. In (**d**) we show the variations of $p(\varepsilon )$ for fixed values of $(\alpha _A/d,\eta ) = (1,1)$ and $w_0 = 1,5,10$. In all figures, the black continuous lines are given by Eq. (4) with $(q,\beta _q)$ given by Eqs. (5,  6) respectively. *Insets:* $\ln _q$-linear representation of the same data; the slopes of the straight lines precisely yield the corresponding values of ($-\beta _q$). The simulations were averaged over $10^3$ realisations for $N=10^{5}$.

We know that the signature of the Boltzmannian systems is the presence of exponentials and Gaussians distributions. Similarly, the nonextensive systems based on the entropy $S_q$ can be recognised by the emergence of *q*-exponentials and *q*-Gaussians distributions. We have here found that, independent of the choice of $(d, \alpha _A, \alpha _G, w_0,\eta )$, the *‘energy’ distribution* $p(\varepsilon )$ associated with the network is *invariably* well fitted by the following *q*-exponential:

4 $$\begin{aligned} p_q(\varepsilon ) =\frac{e_q^{-\beta _{q} \varepsilon }}{Z_q}, \end{aligned}$$

where $p_q(\varepsilon )$ represents the generalisation, within nonextensive statistical mechanics, of the BG energy weight with $\varepsilon$, $\beta _q$ and $Z_q$ playing respectively the roles of energy, inverse temperature and normalisation factor (see Fig. 3). Note that, when $q\rightarrow 1$, we do recover the standard Boltzmann distribution since $e_1^{-\beta _1 \varepsilon }\equiv e^{-\beta \varepsilon }$. This result exhibits an interesting emergence of correspondence between a random network geometric problem and a particular case within generalised thermostatistics. This fact definitively reminds the Kasteleyn–Fortuin theorem^33^, which establishes an important isomorphism between the bond-percolation geometric problem and the $q_{Potts} \rightarrow 1$ limit of the $q_{Potts}$-state Potts ferromagnet.

For all $(d, \alpha _A, \alpha _G, w_0, \eta )$, we found that:

5 $$\begin{aligned} q= & {} {\left\{ \begin{array}{ll} \frac{4}{3} &{}\text{ if }\; 0\le \frac{\alpha _A}{d} \le 1\\ \frac{1}{3}\,e^{1-\alpha _A/d} + 1 &{}\text{ if }\; \frac{\alpha _A}{d} > 1 \end{array}\right. } \end{aligned}$$

6 $$\begin{aligned} \beta _q= & {} {\left\{ \begin{array}{ll} \displaystyle \beta _{q_0} &{}\text{ if }\; 0 \le \frac{\alpha _A}{d} \le 1\\ \displaystyle (\beta _{q_0} - \beta _{q_\infty })\,e^{2(1-\alpha _A/d)} +\beta _{q_\infty }&{}\text{ if }\; \frac{\alpha _A}{d} > 1 \end{array}\right. } \end{aligned}$$

with $\beta _{q_0} \simeq (-10.81 e^{-1.36\eta } + 6.04)/w_0$ and $\beta _{q_\infty } \simeq (-4.81 e^{-1.22\eta } + 2.56)/w_0$ . As can be seen, *q* does not depend on $(\eta ,w_0)$, but only on the scaled variable $\alpha _A/d$. In contrast, $\beta _q$ is less universal and depends on all three parameters $(w_0,\eta ,\alpha _A/d)$.

**Figure 4:**
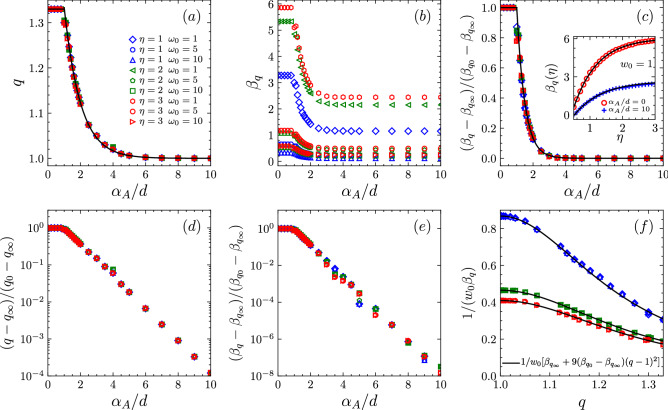
(**a**) *q* as a function of $\alpha _A/d$; *q* is constant in the range $0\le \alpha _A/d \le 1$ ($q_0 = 4/3$) and decreases exponentially with $\alpha _A/d$ for $\alpha _A/d > 1$, down to $q_\infty = 1$ (black solid line). (**b**) $\beta _q$ as a function of $\alpha _A/d$ for $\eta = 1, 2, 3$ and $w_0 = 1, 5,10$, for typical values of $\alpha _A/d$; $\beta _q$ increases with $\eta$ and decreases with $w_0$ and $\alpha _A/d$. (**c**) By plotting $(\beta _{q} - \beta _{q_\infty })/(\beta _{q_0} - \beta _{q_\infty })$, all curves collapse and exponentially decrease with $\alpha _A/d >1$ (black straight line). Inset: $\beta _{q_0}$ and $\beta _{q_\infty }$ exponentially vary with $\eta$; $\beta _{q_\infty }$ was estimated by fixing $\alpha _A/d =10$. In (**d**,**e**) we present $\log$-linear representations of the same data as in (**a**,**c**) respectively, exhibiting the exponential dependence of both *q* and $\beta _q$ on $\alpha _A/d$, when $\alpha _A/d \ge 1$. (**f**) By eliminating the variable $\alpha _A/d$, we show $1/(w_0\beta _q)$ as functions of *q* for the same set of data shown in the previous plots; *q* is related with $1/(w_0\beta _q)$ through the equation $1/w_0[\beta _{q_\infty } + 9(\beta _{q_0} - \beta _{q_\infty })(q-1)^2]$ that is valid for all values of $(w_0,\eta$). For very large values of $\alpha _A/d$ and the extreme regions $\eta \rightarrow 0$ and $\eta \rightarrow \infty$, the numerical precision needed to attain the stationary-state distribution is too high for our present computational effort and further analysis would be needed, which is out of the present scope.

In Fig. 4a,d we show the numerical results for the index *q* as function of $\alpha _A/d$. This result is consistent with^19,20^, where the behaviour of *q* characterises the existence of three regimes. As can be seen, *q* is constant and equal to 4/3 in the range $0 \le \alpha _A/d \le 1$. This interval describes the regime of strong-long-range interactions characterised by the highest value of *q*. In the interval $1< \alpha _A/d \lesssim 5$ we have the moderately-long-range interaction regime, characterised by *q* smaller than 4/3 but still greater than 1. In this regime *q* displays no abrupt transition from 4/3 to 1 but instead it decreases exponentially with $\alpha _A/d$ through the function $e^{1-\alpha _A/d}$^19,20^. This behaviour exhibits that the BG regime was not yet reached. In the last regime, $\alpha _A/d \gtrsim 5$, the Boltzmannian-like regime finally emerges and $q = 1$. Similar results for *q* were found in^17^ for a gas-like network model. In the Fig. 4b,c,e we show similar results for the parameter $\beta _q$ which equals $\beta _{q_0}$ in the range $0\le \alpha _A/d\le 1$ and, then, exponentially decreases with $\alpha _A/d$; $\beta _q$ increases with $\eta$ and decreases with $w_0$. However, if we plot $(\beta _{q} - \beta _{q_\infty })/(\beta _{q_0} - \beta _{q_\infty })$, all curves collapse as a function of $\alpha _A/d$. Moreover, we verify in Fig. 4f that the effective ‘temperature’ *decreases* with increasing *q*. There is no thermodynamical prescription which would impose that. In^30^, for instance, both possibilities are in fact observed.

## Discussion

All these results strongly suggest that the ‘energy’ distribution $p(\varepsilon )$ of the network is given by the very same expression which *q*-generalises the Boltzmann–Gibbs weight when it is the nonadditive entropy $S_q$ which is optimised. Naturally, since the present study is numerical, we can not exclude very minor corrections due to high-order metric-topological terms. The BG limit is rapidly reached when $\alpha _A/d\gtrsim 5$. Not less important, *q* and $\beta _q$ depend on $\alpha _A$ and *d* only through the ratio $\alpha _A/d$; also, interestingly enough, none of them depends on $\alpha _G$. The fact that *q* depends *only* on $\alpha _A/d$ means that this ratio uniquely determines the entropic nonadditivity universality class. The quantity $\beta _q$ also depends on $\eta$ and $w_0$. Consistently, $w_0$, which characterises the width of the stretched-exponential distribution *P*(*w*), plays here the same role as *T* in usual thermal BG problems. This seemingly is the first time that, in a complex network, we identify a parameter which plays the role of an external parameter that we may fix at will, similarly to the manner in which we fix, in BG statistical mechanics, the temperature at which the thermally equilibrated system is placed. In all previous connections with random networks^16–22^, $\beta _q$ (sometimes noted $1/\kappa _q$) was univocally related to *q*. A single value for $\beta _q$ for a given value of *q* is analogous to traditional critical points in BG statistics. In our present case, we have, for a fixed value of *q*, the freedom of making $\beta _q$ to vary, like *T* in BG thermal statistics.

The present results strongly support the conjecture of existence of a neat correspondence between geometrical random network (asymptotically) scale-invariant problems and the present specific class of many-body models within nonextensive statistical mechanics, constructed upon nonadditive entropies. This is analogous to the Kasteleyn-Fortuin theorem for the $q_{Potts}$-state Potts model, whose $q_{Potts}\rightarrow 1$ limit rigorously corresponds to the bond percolation problem^33^, and also to the de Gennes celebrated isomorphism for the *n*-vector ferromagnetic model, whose $n\rightarrow 0$ limit precisely corresponds to the self-avoiding random walk^34^, which constitutes a pillar in polymer physics. It is possible to think of a variety of applications of connections of the present kind, for example the maintaining budgets to be distributed among cities connected within a large regional network of roads. Each city could, for instance, receive a support proportional to the sum of the widths of the roads arriving to it.

## Methods

To calculate the relevant properties of our network model in a statistically relevant way, we used 1000 independent realisations within the standard Monte Carlo method to generate different instances of the our network. The network size was set to be $N=10^5$. All simulations were obtained through independent codes in C. To generate random numbers from the stretched exponential distribution we used the boost library available in https://www.boost.org/. Logarithmic binning was used to generate the histogram of the energies using Python packages of the *numpy* library.

## Data Availability

The data and the codes that support all the results within this paper are available from the corresponding authors upon request.
